# Pim kinase inhibitor co-treatment decreases alternative non-homologous end-joining DNA repair and genomic instability induced by topoisomerase 2 inhibitors in cells with FLT3 internal tandem duplication

**DOI:** 10.18632/oncotarget.28042

**Published:** 2021-08-31

**Authors:** Mario Scarpa, Shivani Kapoor, Eric S. Tvedte, Kshama A. Doshi, Ying S. Zou, Prerna Singh, Jonelle K. Lee, Aditi Chatterjee, Moaath K. Mustafa Ali, Robin E. Bromley, Julie C. Dunning Hotopp, Feyruz V. Rassool, Maria R. Baer

**Affiliations:** ^1^University of Maryland Greenebaum Comprehensive Cancer Center, Baltimore, MD, USA; ^2^Department of Medicine, University of Maryland School of Medicine, Baltimore, MD, USA; ^3^Institute for Genome Sciences, Baltimore, MD, USA; ^4^Department of Pathology, University of Maryland School of Medicine, Baltimore, MD, USA; ^5^Department of Microbiology and Immunology, University of Maryland School of Medicine, Baltimore, MD, USA; ^6^Department of Radiation Oncology, University of Maryland School of Medicine, Baltimore, MD, USA; ^7^Veterans Affairs Medical Center, Baltimore, MD, USA

**Keywords:** FLT3 internal tandem duplication, Pim kinase, alternative non-homologous end-joining DNA repair, genomic instability, topoisomerase 2 inhibitors

## Abstract

Acute myeloid leukemia (AML) with fms-like tyrosine kinase 3 internal tandem duplication (FLT3-ITD) relapses with new chromosome abnormalities following chemotherapy, implicating genomic instability. Error-prone alternative non-homologous end-joining (Alt-NHEJ) DNA double-strand break (DSB) repair is upregulated in FLT3-ITD-expresssing cells, driven by c-Myc. The serine/threonine kinase Pim-1 is upregulated downstream of FLT3-ITD, and inhibiting Pim increases topoisomerase 2 (TOP2) inhibitor chemotherapy drug induction of DNA DSBs and apoptosis. We hypothesized that Pim inhibition increases DNA DSBs by downregulating Alt-NHEJ, also decreasing genomic instability. Alt-NHEJ activity, measured with a green fluorescent reporter construct, increased in FLT3-ITD-transfected Ba/F3-ITD cells treated with TOP2 inhibitors, and this increase was abrogated by Pim kinase inhibitor AZD1208 co-treatment. TOP2 inhibitor and AZD1208 co-treatment downregulated cellular and nuclear expression of c-Myc and Alt-NHEJ repair pathway proteins DNA polymerase θ, DNA ligase 3 and XRCC1 in FLT3-ITD cell lines and AML patient blasts. ALT-NHEJ protein downregulation was preceded by c-Myc downregulation, inhibited by c-Myc overexpression and induced by c-Myc knockdown or inhibition. TOP2 inhibitor treatment increased chromosome breaks in metaphase spreads in FLT3-ITD-expressing cells, and AZD1208 co-treatment abrogated these increases. Thus Pim kinase inhibitor co-treatment both enhances TOP2 inhibitor cytotoxicity and decreases TOP2 inhibitor-induced genomic instability in cells with FLT3-ITD.

## INTRODUCTION

Internal tandem duplication (ITD) of the *fms*-like tyrosine like kinase 3 (FLT3) receptor tyrosine kinase is present in acute myeloid leukemia (AML) cells of 30 percent of patients [1], resulting in constitutive and aberrant FLT3 signaling [2]. Remission induction chemotherapy including the nucleoside analog cytarabine (AraC) and an anthracycline and/or other topoisomerase 2 inhibitor remains first-line therapy for AML [3]. While patients with AML with FLT3-ITD generally achieve remission with chemotherapy, they relapse rapidly [1] and AML cells frequently have new structural chromosome abnormalities at relapse [4], consistent with a role of genomic instability in FLT3-ITD AML progression and relapse. FLT3 inhibitors have been incorporated into treatment of AML with FLT3-ITD, but their efficacy is frequently limited and transient and treatment outcomes remain suboptimal [5]. Therefore drugs targeting other molecules in FLT3-ITD signaling pathways are also being explored [5].

The Pim kinases, Pim-1, Pim-2 and Pim-3, are a family of oncogenic serine/threonine kinases that contribute to regulation of multiple key cellular proteins, including the transcription factor c-Myc [6]. Pim-1 is transcriptionally upregulated downstream of FLT3-ITD [7] and also phosphorylates and stabilizes FLT3 and thereby promotes FLT3 signaling in a positive feedback loop in cells with FLT3-ITD [8, 9]. Inhibition of Pim kinases is therefore an attractive therapeutic approach in AML with FLT3-ITD. Pan-Pim kinase inhibitors are being developed, and several have proceeded to early phase clinical testing [10–12]; the most recent is TP-3654 (https://clinicaltrials.gov/). We previously showed that concurrent treatment with a Pim kinase inhibitor enhances induction of apoptosis by topoisomerase 2 inhibitor chemotherapy drugs in cells with FLT3-ITD [13]. Mechanistically, co-treatment with Pim kinase inhibitor increases topoisomerase 2 inhibitor induction of reactive oxygen species (ROS) and DNA double-strand breaks (DSBs) [13]. Of note, increased apoptosis occurs without changes in cell cycle distribution in residual cells, and without cell cycle arrest [13].

DNA DSBs are repaired by homologous recombination (HR) or classical (C-) or alternative (Alt-) non-homologous end-joining (NHEJ) [14]. HR, mediated by BRCA1, BRCA2 and RAD51 and occurring in the S and G2 phases of the cell cycle, uses the complementary sister chromatid strand as a template for repair and is therefore highly accurate and error-free [14]. C-NHEJ is initiated by the Ku proteins Ku70/80, followed by recruitment of the DNA-dependent protein kinase catalytic subunit (DNA-PKc), and mediates repair by directly ligating the ends of DSBs, creating the potential for small deletions, insertions and other structural errors at repair sites [14, 15]. Alt-NHEJ, mediated by poly (ADP-ribose) polymerase (PARP) 1, DNA polymerase θ, DNA ligase 3 and its stabilizing co-factor X-ray repair cross-complementing protein 1 (XRCC1) [16], occurs via microhomology and is therefore characterized by large DNA deletions and chromosomal translocations, leading to genomic instability [14, 15].

Cells with FLT3-ITD exhibit increased generation of ROS and increased DNA DSBs, as well as decreased Ku protein levels and increased DNA ligase 3 and PARP1 protein levels and Alt-NHEJ repair activity [17, 18]. The new structural chromosome changes frequently seen in AML cells with FLT3-ITD at relapse [4] are attributed at least in part to genomic instability caused by error-prone Alt-NHEJ repair of the increased DNA DSBs present in cells with FLT3-ITD [17, 18], and genomic instability is thought to contribute to relapse [4, 19].

Here we sought to determine whether the accumulation of DNA DSBs seen in cells with FLT3-ITD treated with Pim kinase inhibitor in conjunction with topoisomerase 2 inhibitors, relative to topoisomerase 2 inhibitors alone, is due at least in part to decreased DNA DSB repair. Additionally, given that DNA DSB repair occurs primarily via Alt-NHEJ in cells with FLT3-ITD, we hypothesized that the increase in DNA DSBs seen in FLT3-ITD cells treated with Pim kinase and topoisomerase 2 inhibitors, relative to topoisomerase 2 inhibitors alone, may be due to decreased Alt-NHEJ DNA repair, with consequent decrease in genomic instability.

## RESULTS

### Topoisomerase 2 inhibitor treatment upregulates Alt-NHEJ in cells with FLT3-ITD, and Pim inhibitor co-treatment abrogates Alt-NHEJ upregulation

Ba/F3-ITD cells, transfected with human FLT3-ITD, with stably integrated DSB repair reporters DR-GFP, EJ2-GFP or EJ5-GFP transduced with lentivirus expressing mCherry-tagged I-SceI (Supplementary Figure 1A) were treated with the topoisomerase 2 inhibitor daunorubicin and/or the pan-Pim kinase inhibitor AZD1208, or DMSO control, and harvested at 24 and 36 hours. Repair activity was measured by quantifying GFP-positive cells by flow cytometry. Drug treatments did not alter the percentage of mCherry-positive (mCh+) Ba/F3-ITD cells expressing DR-GFP (Supplementary Figure 1B), EJ2-GFP or EJ5-GFP, measured by flow cytometry, demonstrating that I-SceI levels were not altered by drug treatments.

**Figure 1:**
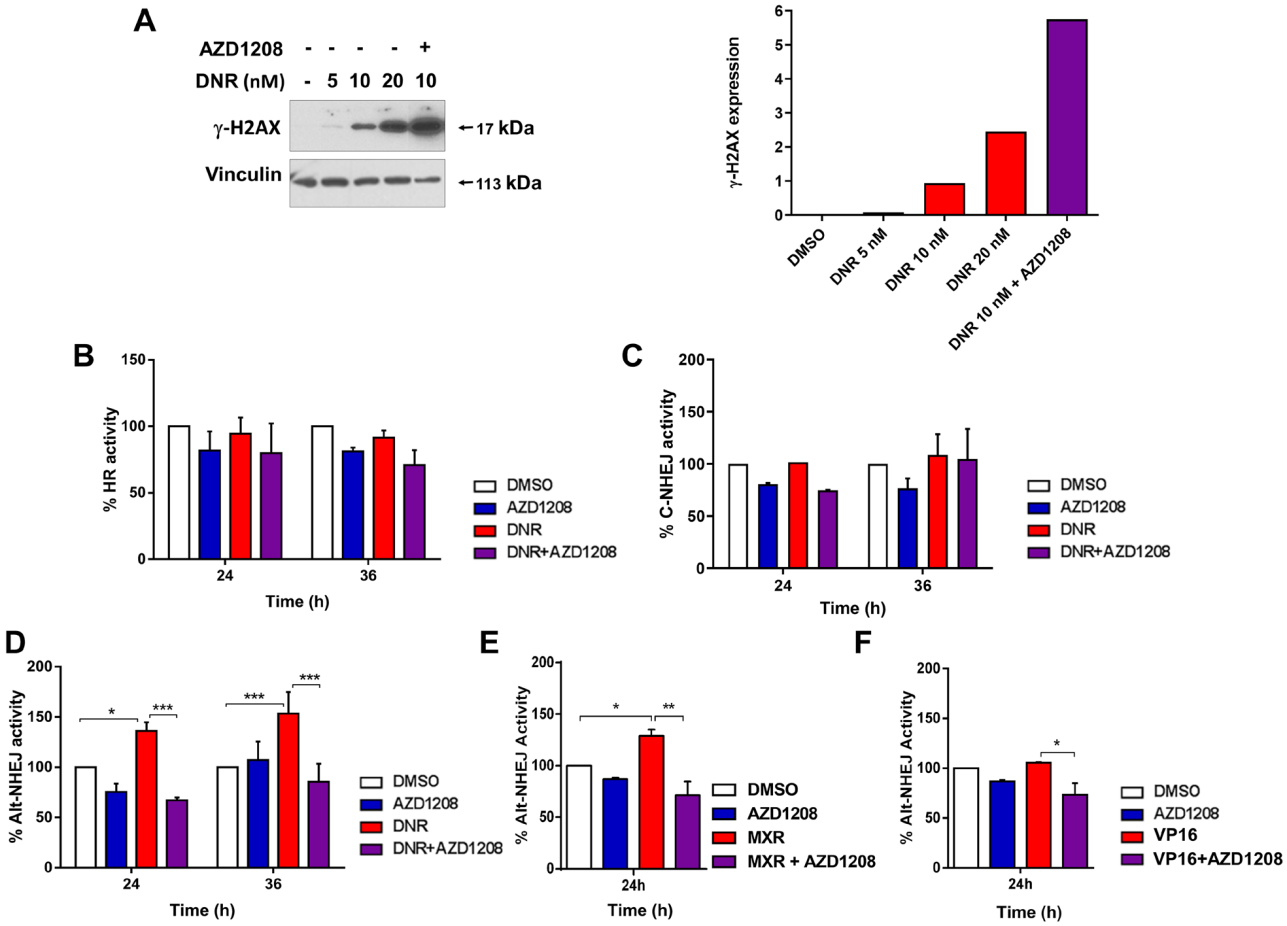
Topoisomerase 2 inhibitor treatment upregulates Alt-NHEJ, but not HR or C-NHEJ, DNA DSB repair activity in cells with FLT3-ITD, and Pim inhibitor co-treatment abrogates Alt-NHEJ upregulation. (**A**) Concurrent treatment with AZD1208 increases DNA damage in Ba/F3-ITD cells. Ba/F3-ITD cells were treated for 36 hours with 0, 5, 10 and 20 nM daunorubicin and 10 nM daunorubicin with 1 μM AZD1208. Immunoblot is shown on the left, and data are graphed on the right. (**B**–**F**) Effects of topoisomerase inhibitors and of topoisomerase inhibitors and AZD1208 on DNA repair. Ba/F3-ITD cells with stable integration of the DNA DSB repair reporters pDRGFP, pimEJ5GFP and EJ2GFP-puro, used to measure HR, C-NHEJ and Alt-NHEJ repair activities, respectively, were transduced with I-SceI lentivirus and treated with (B–D). 10 nM DNR, (E) 30 nM MXR or (F) 20 nM VP-16, and/or 1 μM AZD1208. HR (B), C-NHEJ (C) or Alt-NHEJ (D, E, F) repair activity was measured as percentage of GFP+ cells by flow cytometry, normalized to levels with DMSO control treatment, defined as 100%. Means + S.E.M. of triplicate experiments are shown. ^*^ *p* < 0.05, ^**^ *p* < 0.005, ^***^ *p* < 0.0005.

As described previously [13], treatment with daunorubicin increased γ-H2AX levels [20], indicating induction of DNA damage, in Ba/F3-ITD cells, and co-treatment with the Pim-kinase inhibitor AZD1208 markedly increased γ-H2AX levels, relative to treatment with daunorubicin alone. Specifically, in Ba/F3-ITD cells treated with daunorubicin for 36 hours, DNA DSBs, measured by γ-H2AX levels , increased with daunorubicin concentration, from 5 to 10 to 20 nM, and, as previously reported [13], co-treatment with the Pim inhibitor AZD1208 at 1 μM increased generation of DNA DSBs, with a marked increase with 10 nM daunorubicin and AZD1208, relative to 10 nM daunorubicin alone, and also relative to 20 nM daunorubicin (Figure 1A). Treatment of Ba/F3-ITD cells with 10 nM daunorubicin did not increase HR or C-NHEJ repair activity (Figure 1B and 1C), but induced a 40% and 50% increase in Alt-NHEJ repair activity at 24 and 36 hours, respectively (Figure 1D). Treatment of Ba/F3-ITD cells with mitoxantrone or etoposide, two other topoisomerase 2 inhibitors used to treat AML, also induced Alt-NHEJ repair activity (Figure 1E and 1F). Concurrent treatment with Pim inhibitor abrogated induction of Alt-NHEJ activity by treatment with all three topoisomerase 2 inhibitors (Figure 1D, 1E and 1F).

To determine whether daunorubicin upregulation of Alt-NHEJ and abrogation by Pim kinase inhibitor co-treatment were specific for cells with FLT3-ITD, Ba/F3-WT cells, transfected with human wild-type FLT3, were studied. Ba/F3-WT cells with stably integrated DSB repair reporters were transduced with lentivirus expressing mCherry-tagged I-SceI, treated with 10 nM daunorubicin and/or 1 μM AZD1208, or DMSO control, and harvested at 24 and 36 hours, as above. γ-H2AX levels increased with concurrent daunorubicin and AZD1208 treatment, relative to daunorubicin alone (Supplementary Figure 2A). Treatment of Ba/F3-WT cells with daunorubicin induced a 50% increase in C-NHEJ activity, and a 20% increase in Alt-NHEJ repair activity at 36 hours, but no change in HR activity, and concurrent treatment with Pim kinase inhibitor decreased daunorubicin induction of both C-NHEJ and Alt-NHEJ repair activity (Supplementary Figure 2B–2D).

### Genome-wide distribution of drug-induced DSBs

We sought to characterize distribution of DNA DSBs following topoisomerase 2 inhibitor treatment with and without Pim kinase inhibitor. To this end, eight paired-end Illumina libraries were prepared from MV4-11 human AML cells with FLT3-ITD in this study (four treatment conditions, daunorubin and/or AZD1208 or DMSO control, each having one DSB-selected library and one negative control library) consisting of 57–87 million paired reads and 10–15 Gbp (Supplementary Table 2). The read mapping rate of DSB-selected libraries to the human genome using HISAT2 was 16–42% and the read mapping rate of negative control libraries was 1–3% (Supplementary Table 1).

**Figure 2:**
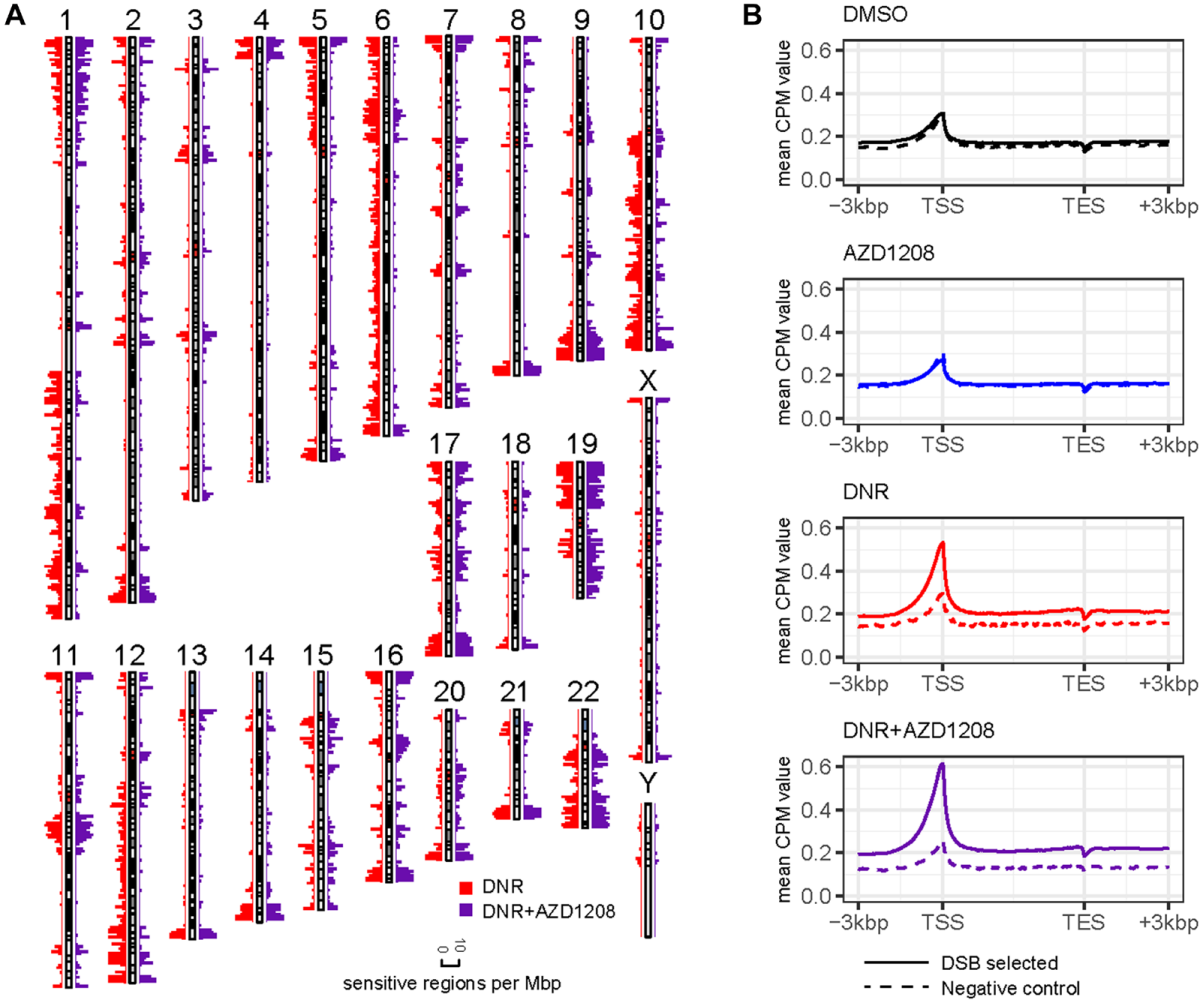
Genome-wide distribution of DSBs by DSB-seq. Illumina reads from DSB-seq and negative control libraries were mapped to the human genome assembly GRCh38 using HISAT2 and genome-wide distribution was characterized using deepTools. (**A**) Human genome sensitivity landscape in MV4-11 cells after treatment with daunorubicin with and without co-treatment with AZD1208. The spatial distribution of sensitive regions is displayed as counts per 1 Mbp of the GRCh38 human genome assembly. (**B**) Distribution of DSB-seq reads mapped to human coding gene regions. After normalizing a non-redundant set of genes to a uniform length, mean mapping values were calculated for all DSB-seq datasets and negative controls. Values are expressed as counts per million mapped reads (CPM).

Human genome regions sensitive to drug-induced DSBs were characterized by enrichment of DSB-seq reads in the daunorubicin and daunorubicin plus AZD1208 treatment conditions relative to DMSO. Enrichment scores were calculated according to the hypergeometric probability distribution. Drug-sensitive regions were observable at multiple genome window scales, supporting valid enrichment of DSBs (Supplementary Figure 3). Using 100 kbp sliding windows across the human genome, a total of 4,761 regions (15%) were sensitive to daunorubicin and 4,182 regions (13%) were sensitive to daunorubicin and AZD1208 co-treatment (Figure 2A). Drug-sensitive regions were distributed across the genome and generally had similar spatial profiles with or without AZD1208 co-treatment (Figure 2A). Of note, however, multiple chromosome regions (e.g., 1q, 5p, 6, 10q, 12q) showed sensitivity to daunorubicin alone but not with AZD1208, suggesting that co-treatment might possibly attenuate DSBs in specific chromosome regions.

DSBs were prevalent in human coding gene regions, particularly at transcriptional start sites (TSS) (Figure 2B). In MV4-11 cells treated with DMSO or with AZD1208 alone, DSB-seq read mapping profiles were similar in the DSB-selected and negative control libraries, possibly indicating (a) spontaneous DNA damage at TSS in untreated cells and/or (b) nonspecific biotinylation labeling during library preparation. DSB peaks were qualitatively more prominent at TSS in MV4-11 cells treated with daunorubicin or with daunorubicin and AZD1208, suggesting increased induction of DNA breaks (Figure 2B).

### Combined Pim and topoisomerase 2 inhibitor treatment downregulates Alt-NHEJ repair pathway proteins in cell lines and AML patient cells with FLT3-ITD, and markedly downregulates c-Myc

To study the mechanism(s) by which Pim inhibitor abrogates topoisomerase 2 induction of Alt-NHEJ activity in cells with FLT3-ITD, we measured levels of the Alt-NHEJ proteins PARP1, DNA polymerase θ, DNA ligase 3 and its stabilizing co-factor XRCC1 [18] after treatment with daunorubicin and/or AZD1208, or DMSO control, and we also measured levels of c-Myc protein, which has been shown to regulate PARP1 and DNA ligase 3 transcription [21].

**Figure 3:**
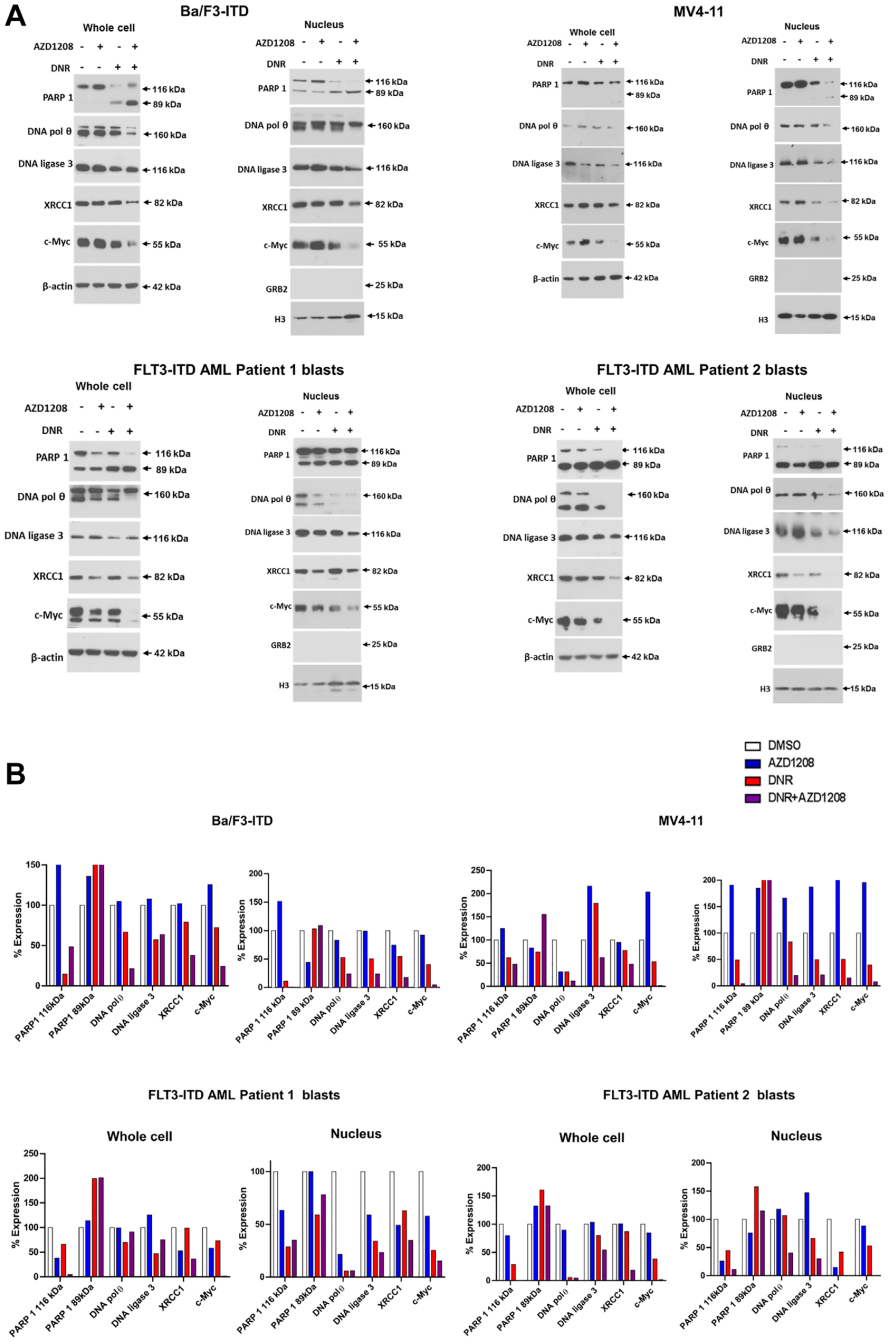
Effect of daunorubicin and/or Pim kinase inhibitor treatment on levels of Alt-NHEJ pathway proteins and c-Myc protein in cells with FLT3-ITD. Levels of PARP1, DNA polymerase θ, DNA ligase 3, XRCC and c-Myc protein were measured by immunoblotting in whole cell and nuclear extracts prepared from Ba/F3-ITD and MV4-11 cells and blood blasts from two patients with AML with FLT3-ITD treated with 10 nM DNR and/or 1 μM AZD1208, or DMSO control, for 36 hours. (**A**) Immunoblots. (**B**) Graphic representation of whole cell and nuclear protein levels, normalized to β-actin protein levels.

Ba/F3-ITD and MV4-11 cells and blood blasts from two patients with AML with FLT3-ITD were cultured for 36 hours with daunorubicin and/or AZD1208, or DMSO control, and levels of PARP1, DNA polymerase θ, DNA ligase 3, XRCC1 and c-Myc protein were measured in whole cell and nuclear lysates (Figure 3A). Data are shown graphically in Figure 3B. Treatment with AZD1208 had no effect on levels of these proteins, relative to DMSO control, while treatment with daunorubicin alone decreased levels of most proteins, and AZD1208 and daunorubicin combination treatment decreased levels of all proteins, relative to daunorubicin alone, in whole cell and nuclear lysates, with decrease in nuclear levels most prominent, and marked decrease in c-Myc levels.

### Topoisomerase 2 inhibitor and Pim kinase inhibitor co-treatment downregulates c-Myc via increased proteasomal degradation

**Figure 4:**
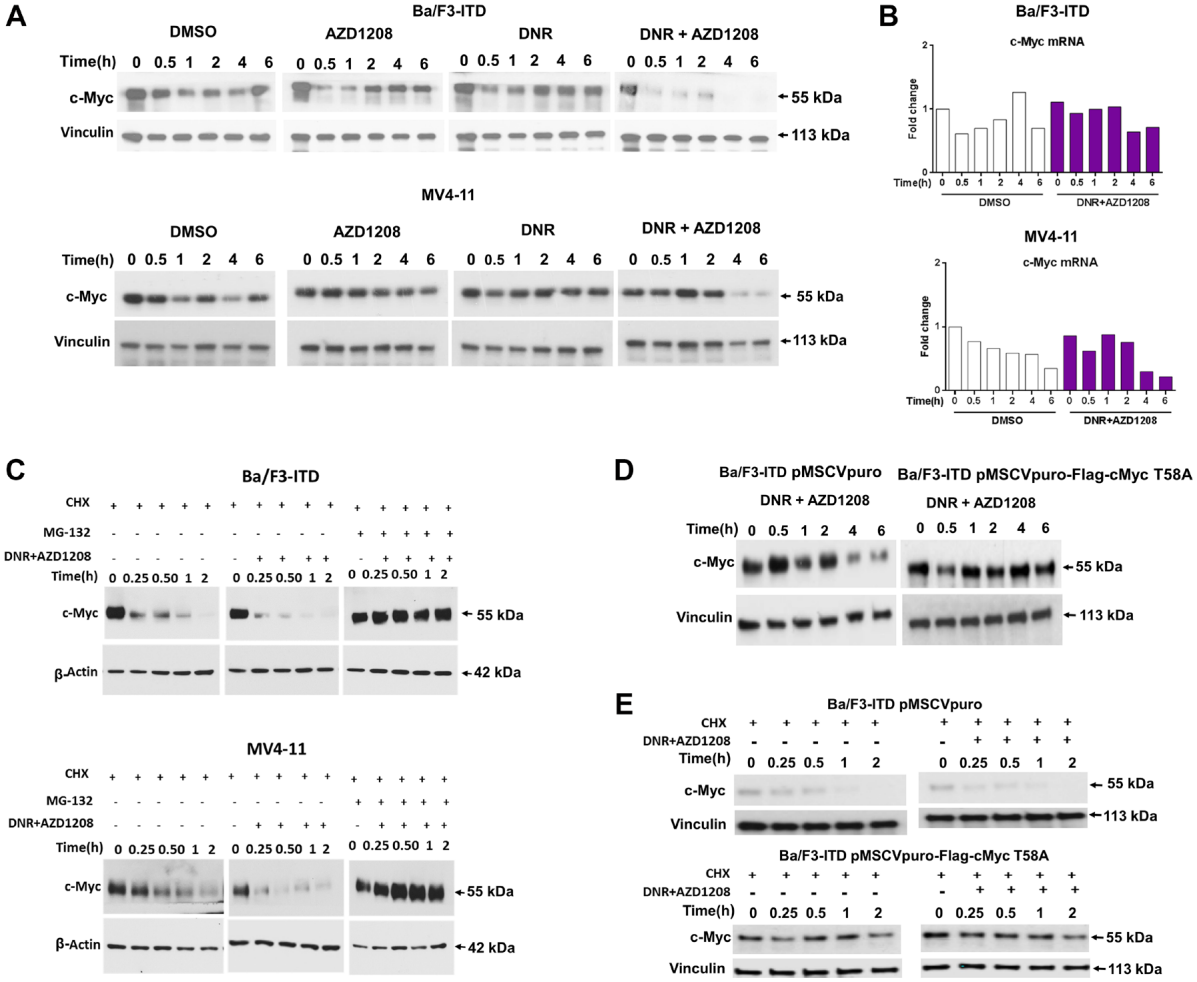
Topoisomerase 2 inhibitor and Pim kinase inhibitor co-treatment downregulates c-Myc through increased proteasomal degradation. Ba/F3-ITD and MV4-11 cells were treated with 10 nM DNR and/or 1 μM AZD1208, or DMSO control, (**A**) c-Myc and vinculin protein levels were measured by immunoblotting in Ba/F3-ITD (top) and MV4-11 (bottom) cells, demonstrating c-Myc downregulation by combination treatment. (**B**) c-Myc mRNA expression at serial time points in Ba/F3-ITD (top) and MV4-11 (bottom) cells normalized to GAPDH mRNA levels and graphed as fold change from time 0 is shown, demonstrating that c-Myc mRNA downregulation did not precede protein downregulation. (**C**) Ba/F3-ITD (top) and MV4-11 (bottom) cells were cultured with 100 μg/mL cycloheximide (CHX) to block new protein translation, and c-Myc protein turnover was measured by immunoblotting at serial time points beginning 60 minutes after addition of CHX. To measure c-Myc protein turnover with DNR and AZD1208 treatment and the effect of proteasomal degradation, DNR and AZD1208 were added 60 minutes into cycloheximide treatment, with and without addition of 20 μM MG-132 30 minutes prior to DNR and AZD1208 treatment, and c-Myc protein levels were measured by immunoblotting at serial time points. c-Myc turnover increased with DNR and AZD1208 treatment, and this increase was abrogated by MG-132 pre-treatment. (**D**) Ba/F3-ITD cells infected with pMSCVpuro-Flag-cMyc T58A plasmid expressing T58A, preventing T58 phosphorylation, or pMSCVpuro empty vector control, were treated with DNR and AZD1208, demonstrating that c-Myc downregulation by DNR and AZD1208 requires T58 phosphorylation. (**E**) Ba/F3-ITD cells infected with pMSCVpuro-Flag-cMyc T58A or pMSCVpuro empty vector control were treated with CHX to inhibit new protein translation and effect of DNR and AZD1208 or DMSO control treatment on c-Myc protein turnover was studied, demonstrating decreased c-Myc turnover in the absence of T58 phosphorylation.

Co-treatment of Ba/F3-ITD cells (Figure 4A, top) and MV4-11 cells (Figure 4A, bottom) with daunorubicin and AZD1208 downregulated c-Myc protein levels at early time points, relative to treatment with each drug alone and with DMSO control. c-Myc protein downregulation was not preceded by mRNA downregulation (Figure 4B).

To determine whether c-Myc protein downregulation was a result of increased proteasomal degradation, Ba/F3-ITD (Figure 4C, top) and MV4-11 (Figure 4C, bottom) cells were pretreated with cycloheximide (CHX) for 60 minutes to block new protein translation, with and without addition of the proteasome inhibitor MG-132 after 30 minutes, then treated with daunorubicin and AZD1208, or DMSO control. In both cell lines, c-Myc protein turnover increased with concurrent AZD1208 and daunorubicin treatment, and this increase was abrogated by pre-treatment with G-132. Thus concurrent AZD1208 and daunorubicin treatment causes post-translational c-Myc downregulation, through enhanced c-Myc proteasomal degradation.

Because Pim-1 phosphorylation of c-Myc mediates a decrease in c-Myc T58 phosphorylation and an increase in c-Myc S62 phosphorylation [6], we hypothesized that Pim inhibitor would increase T58 phosphorylation as a mechanism of post-translational c-Myc downregulation, and would be ineffective in cells infected with pMSCVpuro-Flag-cMyc-T58A plasmid, containing c-Myc with a T58 mutation changing threonine to alanine, preventing phosphorylation. To test this hypothesis, c-Myc levels were measured at serial time points in Ba/F3-ITD cells infected with pMSCVpuro-Flag-cMyc-T58A plasmid or with pMSCVpuro empty vector control treated with daunorubicin and AZD1208. Daunorubicin and AZD1208 co-treatment downregulated c-Myc in Ba/F3-ITD cells infected with pMSCVpuro, but not with pMSCVpuro-Flag-cMyc-T58A (Figure 4D). c-Myc protein turnover was also studied in Ba/F3-ITD cells infected with pMSCVpuro-Flag-cMyc-T58A plasmid or with pMSCVpuro empty vector treated with daunorubicin and AZD1208 or with DMSO control. c-Myc protein was stabilized in cells infected with pMSCVpuro-Flag-cMyc-T58A, and daunorubicin and AZD1208 co-treatment did not increase c-Myc protein turnover (Figure 4E); data are shown graphically in Supplementary Figure 4.

### Expression of Alt-NHEJ repair proteins is regulated by c-Myc

**Figure 5:**
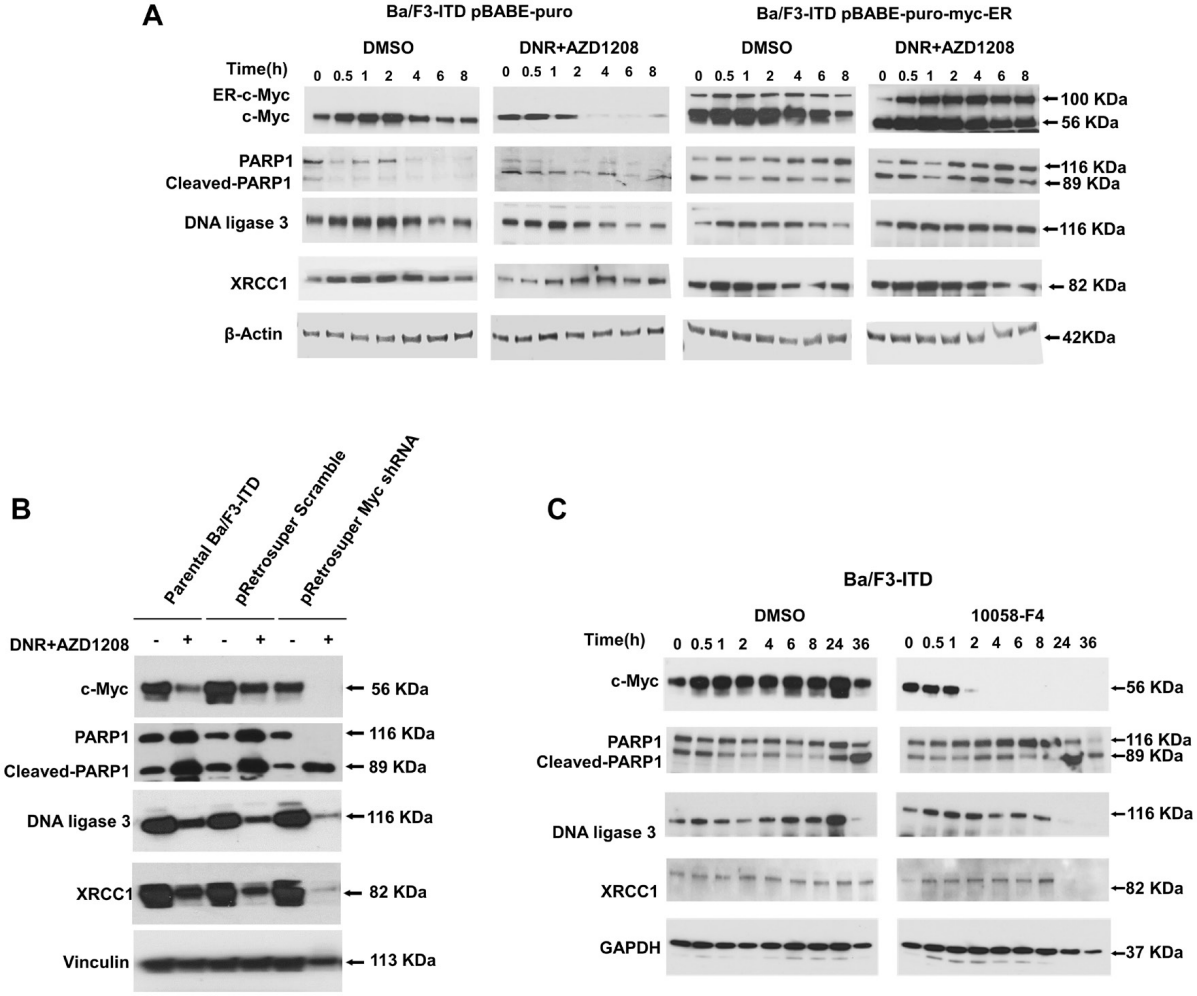
Expression of Alt-NHEJ repair proteins is regulated by c-Myc. (**A**) c-Myc overexpression. Ba/F3-ITD cells infected with pBABEpuro-myc-ER or with pBABEpuro empty vector control and treated with 4-OHT were treated with AZD1208 and daunorubicin and PARP1, DNA ligase 3 and XRCC1 protein levels were measured at serial time points by immunoblotting. Downregulation was seen in cells infected with pBABE-puro, but not with pBABEpuro-myc-ER. (**B**) c-Myc knockdown. Protein levels were measured in parental Ba/F3-ITD cells and Ba/F3-ITD cells infected with pRetrosuper Myc shRNA and pSUPER retro puro Scr shRNA control. c-Myc knockdown markedly increased PARP1, DNA ligase 3 and XRCC1 downregulation by AZD1208 and daunorubicin. (**C**) c-Myc inhibition. Protein levels were measured in Ba/F3-ITD cells treated with the Myc inhibitor 10058-F4. c-Myc protein decreased, followed by decrease in PARP1, DNA ligase 3 and XRCC1 protein.

c-Myc overexpression, knockdown and inhibition were used to study c-Myc regulation of Alt-NHEJ repair protein expression. Concurrent AZD1208 and daunorubicin treatment of Ba/F3-ITD cells infected with pBABEpuro-myc-ER and treated with 4-hydroxytamoxifen (4-OHT) to induce c-Myc overexpression and nuclear translocation, as detailed in Materials and Methods, did not result in downregulation of PARP1, DNA ligase 3 or XRCC1, in contrast to the downregulation seen in Ba/F3-ITD cells infected with pBABEpuro empty vector control treated with AZD1208 and daunorubicin (Figure 5A).

Additionally, c-Myc knockdown markedly increased PARP1, DNA ligase 3 and XRCC1 downregulation by AZD1208 and daunorubicin (Figure 5B). Finally, treatment of Ba/F3-ITD cells with the Myc inhibitor 10058-F4 decreased c-Myc protein levels, followed by decrease in PARP1, DNA ligase 3 and XRCC1 protein levels (Figure 5C).

### Pim kinase inhibition significantly reduces chromosomal instability induced by topoisomerase 2 inhibitor treatment in cells with FLT3-ITD, but not wild-type FLT3

**Figure 6:**
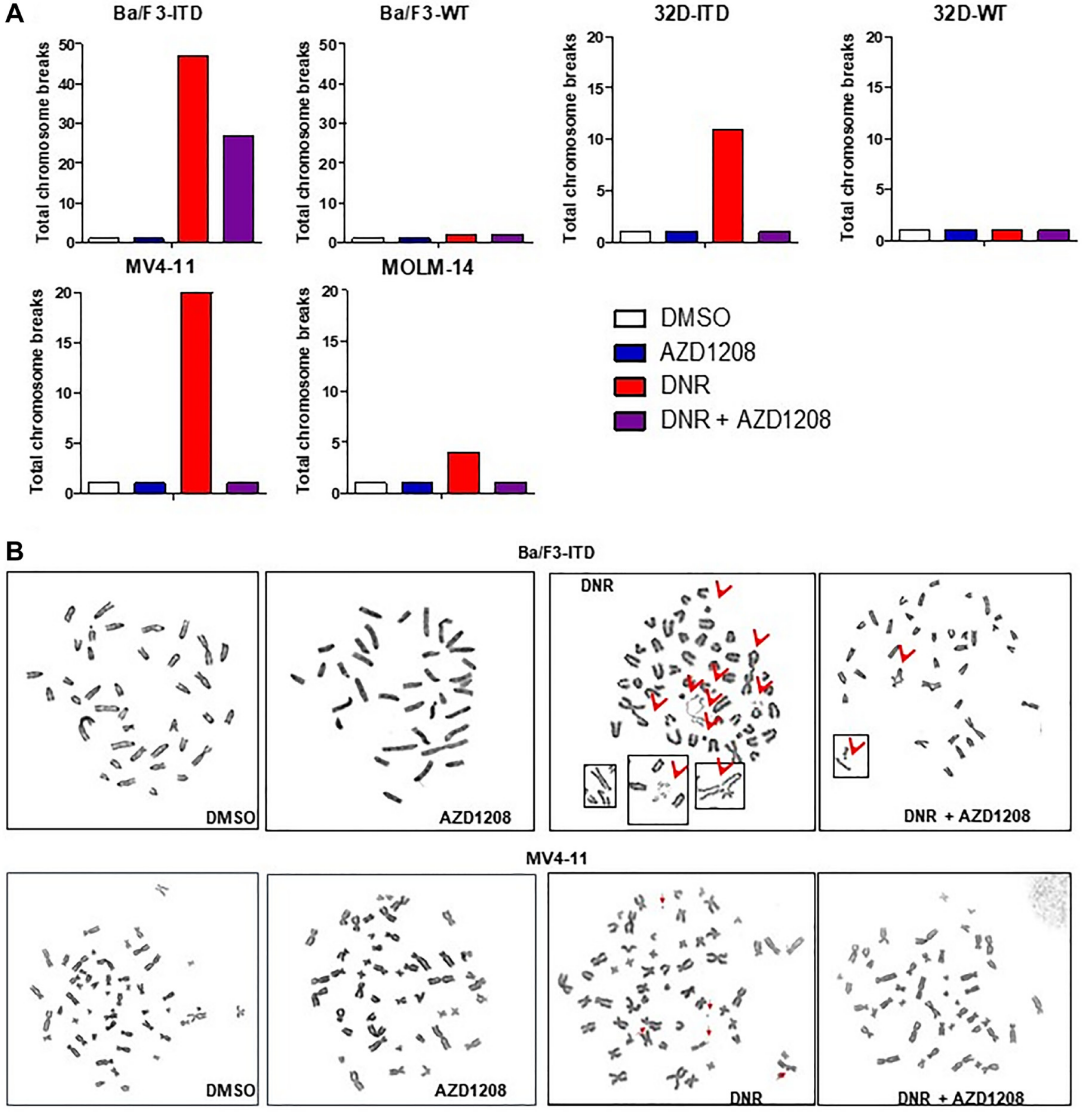
Pim kinase inhibition significantly reduces chromosome breaks induced by topoisomerase 2 inhibitor treatment in cells with FLT3-ITD, but not wild-type FLT3. (**A**) Graphs showing numbers of chromosome breaks. Daunorubicin treatment induced chromosome breaks in Ba/F3-ITD, 32D-ITD, MV4-11 and MOLM-14 cells, with FLT3-ITD, but not in Ba/F3-WT, 32D-WT cells, with wild-type FLT3, and concurrent treatment with Pim kinase inhibitor abrogated or decreased induction of chromosome breaks by daunorubicin treatment in cells with FLT3-ITD. (**B**) Metaphase spreads in Ba-F3-ITD and MV4-11 cells treated with DNR and/or AZD1208 or DMSO control. Induction of increased chromosome breaks by daunorubicin in the absence of concurrent AZD1208 treatment is shown.

Alt-NHEJ repair is associated with increased genomic instability, characterized by increased deletions, insertions and translocations. Having shown that Pim inhibition abrogates induction of Alt-NHEJ by daunorubicin in cells with FLT3-ITD, we studied the effect of daunorubicin and/or AZD1208 treatment on chromosome breaks in Ba/F3-ITD, 32D-ITD, MV4-11 and MOLM-14 cells, with FLT3-ITD, as well as Ba/F3-WT and 32D-WT cells, with wild-type FLT3 (Figure 6). Treatment with daunorubicin for 36 hours induced chromosome breaks in Ba/F3-ITD and 32D-ITD cells, both transfected with human FLT3-ITD, as well as in the human FLT3-ITD AML cell lines MV4-11 (homozygous FLT3-ITD) and MOLM-14 (heterozygous FLT3-ITD), and numbers of chromosome breaks were decreased with concurrent treatment with AZD1208 (Figure 6A). In contrast, daunorubicin treatment did not induce chromosome breaks in Ba/F3-WT or 32D-WT cells, both transfected with human wild-type FLT3, in the absence or presence of AZD1208 (Figure 6A). Metaphase spreads from Ba/F3-ITD and MV4-11 cells with the four treatment conditions are shown in Figure 6B.

## DISCUSSION

FLT3-ITD is present in AML cells of 30% of patients. While patients with AML with FLT3-ITD respond to induction chemotherapy, they relapse rapidly [1]. Addition of the FLT3/multikinase inhibitor midostaurin to initial chemotherapy for FLT3-mutated AML has prolonged patient survival [22], and gilteritinib [23] and other FLT3 inhibitors have activity in relapsed and refractory disease, but outcomes remain suboptimal [5]. New structural chromosome abnormalities are frequently present at relapse of AML with FLT3-ITD [4], implicating genomic instability as a significant driver in the genesis of relapse [4, 19]. Genomic instability in cells with FLT3-ITD is caused by increased generation of ROS and induction and accumulation of DNA DSBs, as well as increased levels of the Alt-NHEJ DNA repair proteins DNA ligase 3 and PARP1, and upregulated error-prone DNA DSB repair activity mediated by Alt-NHEJ [17, 18, 21]. DNA repair by this pathway results in accumulation of chromosome breaks and consequent structural chromosome abnormalities [18]. Notably, we previously reported that c-Myc transcriptionally upregulates levels of the Alt-NHEJ proteins PARP1 and DNA ligase 3, leading to increased error-prone DNA DSB repair activity and genomic instability [21].

The oncogenic serine/threonine kinase Pim-1 is transcriptionally upregulated downstream of FLT3-ITD [8, 9]. We previously showed that combination treatment with a pan-Pim kinase inhibitor increases apoptosis induction by topoisomerase 2 inhibitor chemotherapy drugs, including anthracyclines used in AML remission induction therapy, in cells with FLT3-ITD [13]. Enhanced apoptosis is mechanistically associated with increased oxidative stress and induction and accumulation of DNA DSBs [13]. Here we demonstrate that enhanced DNA damage induction by concurrent Pim kinase inhibitor and topoisomerase 2 inhibitor treatment in cells with FLT3-ITD is associated with, and likely attributable at least in part to, decreased Alt-NHEJ DNA DSB repair, which also decreases genomic instability.

Topoisomerase 2 facilitates DNA replication by generating incisions in both DNA strands, inducing DNA DSBs that aid passage of the replicating DNA duplex [24, 25]. In this process, phosphotyrosyl bonds are formed between topoisomerase 2 and DNA, resulting in formation of transient topoisomerase 2-DNA adducts. Topoisomerase 2 inhibitors, including daunorubicin, mitoxantrone and etoposide, stabilize the topoisomerase 2-DNA adduct, resulting in persistence of DNA DSBs and blockade of replication, leading to cell death [26]. The DNA repair pathways HR, C-NHEJ and nucleotide excision repair (NER) are known to contribute to cell survival following exposure to topoisomerase 2 inhibitors [27–29]. Here we show that DNA DSBs caused by daunorubicin, mitoxantrone or etoposide treatment induce Alt-NHEJ repair activity in cells with FLT3-ITD. In contrast, in cells with wild-type FLT3 DSBs caused by daunorubicin treatment induce C-NHEJ more than Alt-NHEJ. These results are consistent with previous observations that C-NHEJ repair protein levels and activity are downregulated in cells with FLT3-ITD, while Alt-NHEJ protein levels and activity are upregulated [17,18]. Lack of induction of HR in FLT3-ITD or FTL3-WT cells in our study may reflect cell culture conditions, as HR occurs in the S and G2 phases of the cell cycle, and cell culture conditions may not have promoted proliferation [14].

This is, to our knowledge, the first report of Pim kinase regulation of DNA repair in AML cells, and in FLT3-ITD AML cells in particular. Of note, Pim-1 kinase has been shown to regulate DNA repair pathways in other cell types. Pim-1 knockdown or inhibition was found to enhance apoptosis induction by paclitaxel in hormone-refractory prostate cancer cells via decreased C-NHEJ repair associated with decreased ataxia telangiectasia-mutated (ATM) and DNA-PK catalytic subunit (DNA-PKcs) activities, decreased Ku80 levels and nuclear localization, and decreased DNA end-binding of both Ku70 and Ku80 [30]. Additionally, inhibition of Pim-1 kinase in peripheral T-cell lymphoma cells resulted in decreased levels of diverse DNA repair proteins, including ERCC8, which is involved in NER, XRCC2, involved in HR, and XRCC5, involved in C-NHEJ, associated with increased γ-H2AX levels, consistent with increased DNA damage due to decreased levels of DNA repair proteins [31]. Our finding that Pim inhibition abrogates induction of C-NHEJ activity by topoisomerase 2 inhibitor treatment in cells with FLT3-WT is consistent with this finding in prostate cancer and peripheral T-cell lymphoma cells. Of note, daunorubicin treatment did not induce HR activity in Ba/F3-ITD or Ba/F3-WT cells, with FLT3-ITD or FLT3-WT, respectively, in our work. As noted above, HR is cell cycle-specific, occurring in the S and G2 phases of the cell cycle, and lack of induction of HR activity may reflect cell culture conditions perhaps not promoting proliferation. Together with the findings in other cell types detailed above, our data suggest that treatment of diverse cancers with Pim kinase inhibitors in conjunction with chemotherapy drugs that induce DNA damage may be a novel approach to chemosensitization, via decreased DNA damage repair.

This is also, to our knowledge, the first report of Pim kinase regulation of Alt-NHEJ DNA DSB repair. We show for the first time that Pim inhibition abrogates induction of Alt-NHEJ activity by topoisomerase 2 inhibitor treatment in cells with FLT3-ITD. Decreased Alt-NHEJ repair activity likely contributed to the increased DNA damage and increased apoptosis induction that we previously demonstrated in cells with FLT3-ITD treated with Pim Kinase and topoisomerase 2 inhibitors, relative to topoisomerase 2 inhibitors alone [13].

By labeling DSBs in MV4-11 DNA and subsequently sequencing DNA regions proximal to DSBs, we demonstrated genome-wide induction of DSBs by daunorubicin alone or in combination with AZD1208. In addition, gene promoters are apparent hotspots of spontaneous and drug-induced DSBs, a result consistent with previous studies using other topoisomerase 2 inhibitors [32]. Although the results reported here and elsewhere [13] indicate quantitative increases in DSB frequencies per cell in treated FLT3-ITD AML cells, the genome-wide profiles of DSB enrichment are qualitative and therefore cannot be used to directly compare DSBs in cells treated with daunorubicin alone to cells co-treated with AZD1208. Future efforts that simultaneously measure DSB frequencies and their spatial distribution across the genome (e.g., in [33]) will provide accurate quantification of DSBs between treatments.

In addition to the chemosensitization expected from downregulation of any DNA repair mechanism, downregulation of Alt-NHEJ, which is error-prone, has the potential to decrease genomic instability. Alt-NHEJ is upregulated in AML cells with FLT3-ITD [17, 18], and we found here that it is further upregulated by topoisomerase 2 inhibitor treatment of cells with FLT3-ITD. We demonstrated here that abrogation of topoisomerase 2 inhibitor induction of Alt-NHEJ repair in cells with FLT3-ITD by concurrent treatment with a pan-Pim kinase inhibitor is associated with a decrease in induction of chromosome/chromatid breaks, consistent with decreased genomic instability. Given that genomic instability appears to contribute to the frequent rapid relapses seen in patients with AML with FLT3-ITD [4, 19], incorporation of pan-Pim inhibitors into chemotherapy regimens may be beneficial not only for chemosensitization, but also to decrease genomic instability, and decreased genomic instability may have clinical benefit.

**Figure 7:**
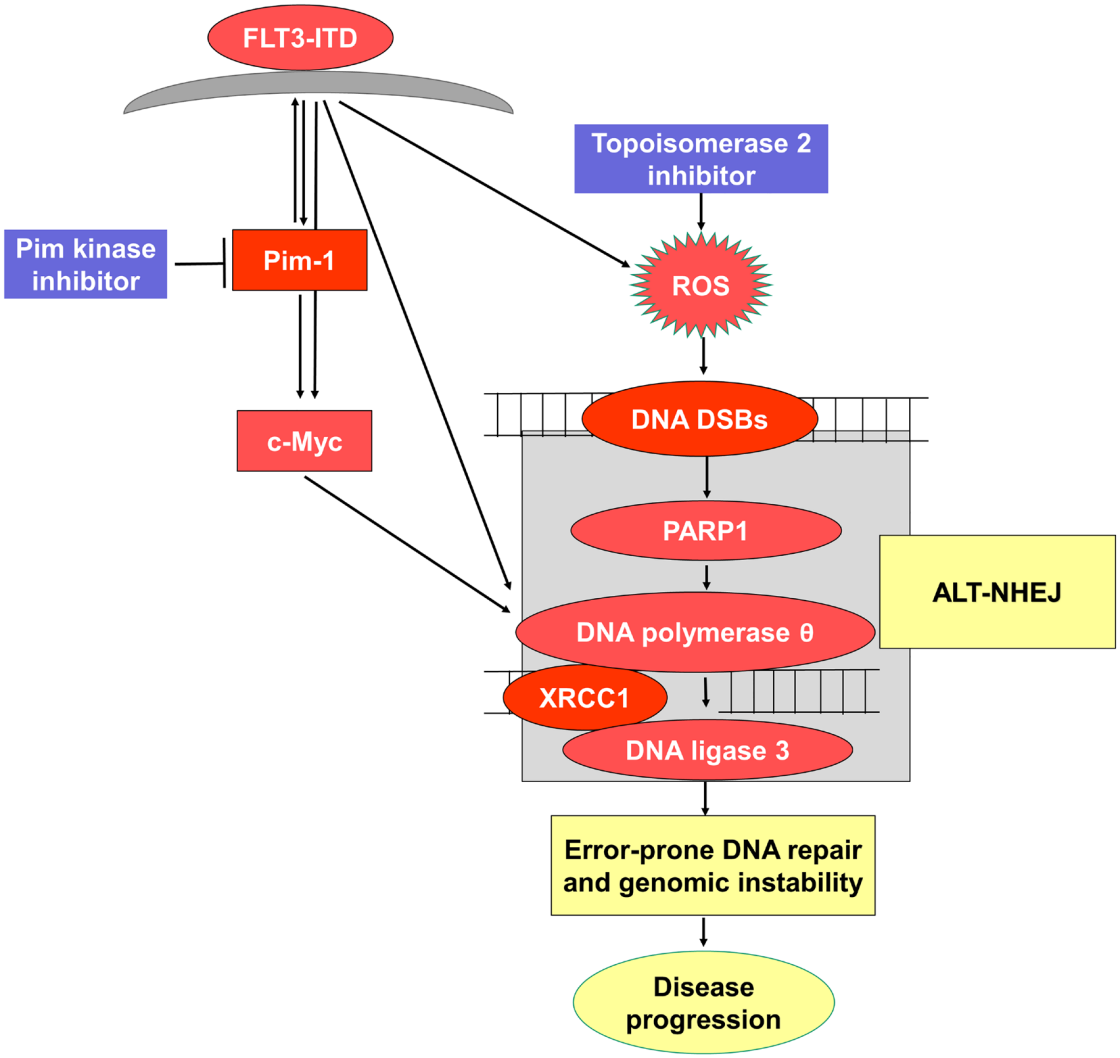
Schematic representation of proposed pathway.

Finally, we sought to determine the mechanism by which concurrent treatment of cells with FLT3-ITD with Pim kinase and topoisomerase 2 inhibitors downregulates Alt-NHEJ DNA repair. We found that concurrent treatment of cells with FLT3-ITD with Pim kinase and topoisomerase 2 inhibitors downregulates c-Myc, that c-Myc downregulation is post-translational, via increased proteasomal degradation, and that the decrease in c-Myc levels results in downregulation of Alt-NHEJ repair proteins (Figure 7). While c-Myc is a Pim-1 substrate, with Pim-1 directly phosphorylating c-Myc and thereby enhancing its stability [6] and altering transcription of c-Myc-regulated genes, treatment of FLT3-ITD-expressing cells with Pim inhibitor alone did not decrease c-Myc levels, while treatment with Pim inhibitor and topoisomerase 2 inhibitor together markedly decreased c-Myc levels. The mechanism of c-Myc downregulation was indeed post-translational, via decreased c-Myc protein stability due to enhanced proteasomal degradation. We previously showed that protein levels of DNA ligase 3 and PARP, two key components of the Alt-NHEJ pathway, are regulated by c-Myc [21].

Pim kinase inhibitors have little single-agent activity in AML *in vitro* [8, 9, 13, 34] or clinically [10], but potently enhance the activity FLT3 inhibitors [8, 9, 34] and topoisomerase 2 inhibitors [13] in AML with FLT3-ITD, and may also enhance the activity of other signal transduction inhibitors and cytotoxic chemotherapeutic agents in other AML subtypes and other hematologic malignancies [12]. This is similar to the effects of the Bcl-2 inhibitor venetoclax, which has limited single-agent clinical activity [35], but potently enhances the clinical efficacy of demethylating agents in AML [36], and was recently approved by the United States Food and Drug Administration for use in these combination regimens. Optimal use of Pim kinase inhibitors in AML and other malignancies remains to be determined.

## MATERIALS AND METHODS

### Cell lines

Ba/F3-ITD, Ba/F3-WT, 32D/ITD, 32D/WT, MV4-11 and MOLM-14 cells were obtained and cultured as described previously [13].

### Patient samples

Pre-treatment blood samples from patients with AML with FLT3-ITD (Supplementary Table 2) were obtained on a University of Maryland Baltimore Institutional Review Board-approved protocol, following written informed consent. Studies were conducted in accordance with the Declaration of Helsinki. Mononuclear cells isolated by density centrifugation over Ficoll-Paque (Sigma-Aldrich, St. Louis, MO, USA) were studied without prior cryopreservation. Cells were cultured in RPMI 1640 medium with 20% fetal bovine serum, without cytokine supplementation.

### Materials

The pan-Pim kinase inhibitor AZD1208, from Tocris Bioscience (Minneapolis, MN, USA), was used at 1 μM based on phase I clinical trial data [10] and on inhibition of BAD phosphorylation at serine 112 as a pharmacodynamic endpoint [37]. The topoisomerase 2 inhibitors daunorubicin (DNR), mitoxantrone (MXR) and etoposide (VP-16) (Sigma-Aldrich) were dissolved in dimethylsulfoxide (DMSO) (less than 0.05 percent) and used at their approximate IC_50_ concentrations of 10, 20 and 30 nM, respectively, in FLT3-ITD cell lines [13]. The protein translation inhibitor cycloheximide (CHX) was from Sigma-Aldrich and the proteasome inhibitor carbobenzoxy-L-leucyl-L-leucyl-L-leucinal (MG-132) from Calbiochem (San Diego, CA, USA). The c-Myc inhibitor 5-[(4-ethylphenyl)methylene]-2-thioxo-4-thiazolidinone (10058-F4), which inhibits c-Myc-Max binding and thereby prevents transcriptional activation of c-Myc targets, was also from Sigma-Aldrich.

### DNA double-strand break repair activity

The DNA DSB repair reporters pDRGFP (Addgene plasmid #26475; https://www.addgene.org/26475), a gift from Dr. Maria Jasin, Memorial Sloan Kettering Cancer Center [38], and pimEJ5GFP and EJ2GFP-puro (Addgene plasmids #44026 and #44025; https://www.addgene.org/44026 and https://www.addgene.org/44025), gifts from Dr. Jeremy Stark, City of Hope National Medical Center [39], were used to measure HR, C-NHEJ and Alt-NHEJ activities, respectively.

The DRGFP plasmid contains a SceGFP cassette that is inactive due to interruption by the presence of a site that can be cleaved by the bacterial endonuclease I-SceI and a 5’ and 3’ truncated form of green fluorescent protein (iGFP). A DSB created in the SceGFP cassette by I-SceI is repaired by HR using iGFP as the template, resulting in activation of GFP expression that can be detected by flow cytometry as a functional measurement of HR activity [40].

The EJ5GFP plasmid consists of a GFP cassette that is separated from its promoter by a puromycin resistance gene flanked by two sites that can be cleaved by I-SceI. Thus, GFP is inactive in the absence of I-SceI, but I-SceI creates two DSBs that undergo end-joining primarily by C-NHEJ, resulting in restoration of GFP expression that can be measured by flow cytometry. Thus GFP is a functional measurement of C-NHEJ activity [40].

The EJ2GFP plasmid consists of a cassette in which an N-terminal tag is fused to the GFP, which is inactive due to disruption by an I-SceI site that is followed by stop codons in all three reading frames. The I-SceI site and the stop codons are flanked by eight nucleotides of microhomology that induce Alt-NHEJ annealing of the I-SceI-induced DSB, resulting in restoration of GFP expression that can be detected by flow cytometry as a functional measurement of Alt-NHEJ activity [40].

The I-SceI plasmid used for lentivirus production (Supplementary Figure 1A), obtained from VectorBuilder (Santa Clara, CA, USA), consists of a coding region for I-SceI and mCherry, both regulated by the common mammalian promoter human elongation factor-1 alpha (EF1a), so that mCherry levels correlate with I-SceI levels post transduction. This plasmid was used to prepare I-SceI lentivirus with the second-generation packaging system.

Ba/F3-ITD cells were nucleofected with circular DR-GFP or linearized EJ5-GFP (restriction enzyme XhoI) or EJ2-GFP (restriction enzyme HpaI), and stable integrants were selected using puromycin (1 μg/ml) treatment. Single-cell clones expressing DR-GFP, EJ5-GFP or EJ2-GFP were obtained by serial dilution in 96-well plates. Reporter cell line clones underwent two rounds of transduction with I-SceI lentivirus in the presence of polybrene (8 μg/ml) to induce DSBs in the repair reporters. Cells transduced with I-SceI were treated with 10 nM daunorubicin, 30 nM mitoxantrone or 20 nM etoposide and/or 1 μM AZD1208, or DMSO control. Functional changes in HR, C-NHEJ and Alt-NHEJ DSB repair activity were measured at 24 and 36 hours by determining percentages of GFP-positive cells by flow cytometry.

Triplicate experiments were performed. Statistical analysis was performed by two-way ANOVA with *post hoc* Bonferroni testing, using GraphPad Prism V.

### DSB labeling and sequencing

Genomic DNA was isolated from MV4-11 cells treated with daunorubicin and/or AZD1208, or DMSO control, with a QIAamp DNA Mini Kit (Qiagen, Germantown, MD, USA). DNA concentration was determined by fluorescence (Qubit dsDNA BR Assay Kit, Invitrogen, Waltham, MA, USA) and size was determined by gel electrophoresis. For each treatment, 5 μg of DNA were biotinylated [41, 42] by 3′-tailing the DSB ends with 4,000 U terminal deoxynucleotidyl transferase (TdT) (Roche, Indianapolis, IN), 0.5 mM dCTP, 5 mM CoCl2, and 0.02 mM Biotin-16-dUTP (Roche) in TdT reaction buffer. Unused biotin was removed by phenol-chloroform extraction and the biotinylated DNA was precipitated twice with ethanol and 2 M ammonium acetate and dissolved in 200 μL of Tris-EDTA (TE) buffer (pH 8.0). The biotinylated DNA was sheared by ultrasonication (E220, Covaris, Woburn, MA, USA) to generate 200–400 bp fragments. Biotinylated fragments were captured with streptavidin beads (Dynabeads kilobaseBINDER kit, Invitrogen). The biotin-streptavidin complex was disrupted by incubation in an elution buffer [10 mM Tris-HCL (pH 7.5), 1 mM EDTA (pH 8.0), and 2.0 M NaCl] at 75°C for four hours. DNA fragments were purified using a QIAquick PCR Purification Kit (Qiagen). Biotinylated tails were removed with 30 U S-1 nuclease (Thermo Fisher) and incubated at 37°C for 30 minutes. The DNA was again purified with the QIAquick PCR Purification Kit. As a negative control, separate libraries were produced for each experimental treatment in the absence of biotinylation. For each library, paired-end reads (2 × 150) were sequenced on an Illumina HiSeq 4000 (San Diego, CA, USA).

### DSB-seq analysis

Low-quality ends and adapter sequences were trimmed from reads by the Institute for Genome Sciences Genomics Resource Center. Reads were mapped to the GRCh38 primary assembly using HISAT2 v.2.1.0 [43] with a maximum insert size of 1 kbp and disallowing spliced alignments. To characterize genome-wide daunorubicin and AZD1208 sensitivity landscapes, the number of DSB-seq reads was calculated for sliding windows using deepTools bamCoverage v.3.1.3 [44, 45], treating each read pair as an individual fragment (-e --samFlagInclude 64) and filtering secondary alignments (--samFlagExclude 256). Regions with enriched DSB-seq reads in treatment conditions relative to DMSO controls were identified using the hypergeometric probability distribution by considering the number of mapped reads in sliding windows in each condition as well as the total number of mapped reads in each condition. To account for multiple hypothesis testing, we corrected *P*-values using the Benjamini-Hochberg method [46]. In order to determine whether the sensitive regions were an artifact, the above analyses were repeated for a series of sliding window sizes (100 bp-1 Mbp).

To identify the distribution of DSB-seq reads in coding regions in the human genome, the number of reads was calculated for 10 bp sliding windows using deepTools as described above with the addition of counts per million (CPM) normalization (--normalizeUsing CPM) for each bin. The gene set from the primary human assembly was downloaded from GENCODE (Release 36) followed by removal of noncoding and redundant transcripts as well as pseudogenes with gffread v. 0.12.4 [47]. The computeMatrix and plotProfile commands of deepTools were used to scale all human gene regions to a common value and calculate mean CPM values along the length of gene regions as well as 3 kbp upstream and downstream.

### Immunoblotting

Whole cell lysates obtained as previously described [13] and nuclear and cytoplasmic lysates obtained using NE-PER™ Nuclear and Cytoplasmic Extraction Reagents (Thermo Fischer Scientific, Waltham, MA, USA) were immunoblotted as previously described [13]. Primary antibodies to PARP1 and c-Myc (Cell Signaling Technology, Danvers, MA, USA), XRCC1 and DNA polymerase θ (Abcam, Cambridge, United Kingdom), DNA ligase 3 (BD Biosciences, San Jose, CA, USA), γ-H2AX (S139) (Millipore Sigma, Burlington, MA, USA), glyceraldehyde 3-phosphate dehydrogenase (GAPDH) (Calbiochem), β-actin (Santa Cruz Biotechnology, Dallas, TX, USA) and vinculin (Sigma-Aldrich) were used. Antibodies to the nuclear protein histone 3 (H3) (Abcam) and the cytoplasmic protein growth factor receptor-bound protein 2 (GRB2) (BD Biosciences) were used as controls in analyses of nuclear and whole cell protein levels. Densitometry was performed with VisionWorks LS Image Acquisition and Analysis Software (UVP, Upland, CA, USA). Protein levels were normalized to β-actin. Triplicate experiments were performed.

### Retroviral infection of Ba/F3-ITD cells

Retroviral packaging and infection of Ba/F3-ITD cells were performed as previously described [48].

Ba/F3-ITD cells were infected with pMSCVpuro-Flag-cMyc-T58A retroviral plasmid (Addgene plasmid #20076; https://www.addgene.org/20076) [49]) from Addgene (Cambridge, MA, USA), containing c-Myc with a point mutation at position 58, changing threonine to alanine and thereby inhibiting phosphorylation, or pMSCVpuro empty vector control (Takara Bio USA, Mountain View, CA, USA).

To induce c-Myc overexpression, Ba/F3-ITD cells were infected with the c-Myc expression vector pBABEpuro-myc-ER (estrogen receptor) (plasmid #19128; https://www.addgene.org/19128) [50] or pBABE-puro empty vector control from Addgene. Myc-ER expression was confirmed by immunoblotting. Ba/F3-ITD cells expressing Myc-ER were cultured with 300 nM 4-hydroxytamoxifen (4-OHT) (Sigma) to activate the myc-ER fusion protein via nuclear translocation of c-Myc [48].

To accomplish c-Myc knockdown, cells were infected with pRetrosuper Myc shRNA (plasmid #15662; https://www.addgene.org/15662) [51] or pSUPER retro puro Scr shRNA control (plasmid #30520; https://www.addgene.org/30520) from Addgene. c-Myc knockdown was confirmed by immunoblotting.

### Real-time reverse transcription polymerase chain reaction (RT-PCR)

RNA isolated from cells from biological triplicate experiments using TRIzol RNA Isolation Reagents (Thermo Fisher Scientific, Waltham, MA, USA) was measured using a NanoDrop™ Lite Spectrophotometer (Thermo Fisher). RNA (500 ng) from each sample was reverse-transcribed using the SuperScript^®^ IV First-Strand Synthesis System (Thermo Fisher). c-Myc, and GAPDH were amplified in duplicate using Power UP SYBR Green Master Mix (Applied Biosystems, Foster City, CA, USA) in the CFX Connect RT-PCR system (Bio-Rad, Hercules, CA, USA), using previously published primers [48]. The ∆Ct method for relative quantification of gene expression was used to determine mRNA expression levels [48]. c-Myc mRNA levels normalized to GAPDH mRNA levels at serial time points were compared to time 0 (pre-treatment) levels, defined as 1.

### Protein turnover and proteasomal degradation

To study protein turnover, cells were treated with 100 μg/mL CHX to inhibit new protein translation, drug treatment was initiated 60 minutes after addition of CHX and protein levels were measured by immunoblotting at serial time points. To measure the effect of proteasomal degradation, protein turnover was studied with and without addition of the proteasome inhibitor MG-132 (20 μM) 30 minutes after addition of CHX and 30 minutes prior to initiation of drug treatment [48].

### Cytogenetic analysis

Cells plated at 1 × 10^5^/ml were treated with drugs for 36 hours in duplicate. Colcemid 0.1 μg/ml was added 75 minutes before harvesting. Cells were then exposed to 0.075 M KCl hypotonic solution for 30 minutes, fixed in 3:1 methanol:acetic acid and stained with 10% Giemsa for 2.5 minutes. Slides were prepared using the Thermotron CDS-5 (Thermotron, Inc., Holland, MI, USA) cytogenetic drying chamber. For each treatment, twenty consecutive analyzable metaphases were analyzed for induction of chromatid breaks and exchanges [52].

## SUPPLEMENTARY MATERIALS


