# Supplementary material for: Pim kinase inhibitor co-treatment decreases alternative non-homologous end-joining DNA repair and genomic instability induced by topoisomerase 2 inhibitors in cells with FLT3 internal tandem duplication
Source: Oncotarget. 2021 Aug 31;12(18):1763–79. doi: 10.18632/oncotarget.28042 (PMC8416564; doi:10.18632/oncotarget.28042)
Supplement: Supplementary file 1 [file oncotarget-12-1763-s001.pdf]

Pim kinase inhibitor co-treatment decreases alternative non-homologous end-joining DNA repair and genomic instability induced by topoisomerase 2 inhibitors in cells with FLT3 internal tandem duplication

SUPPLEMENTARY MATERIALS

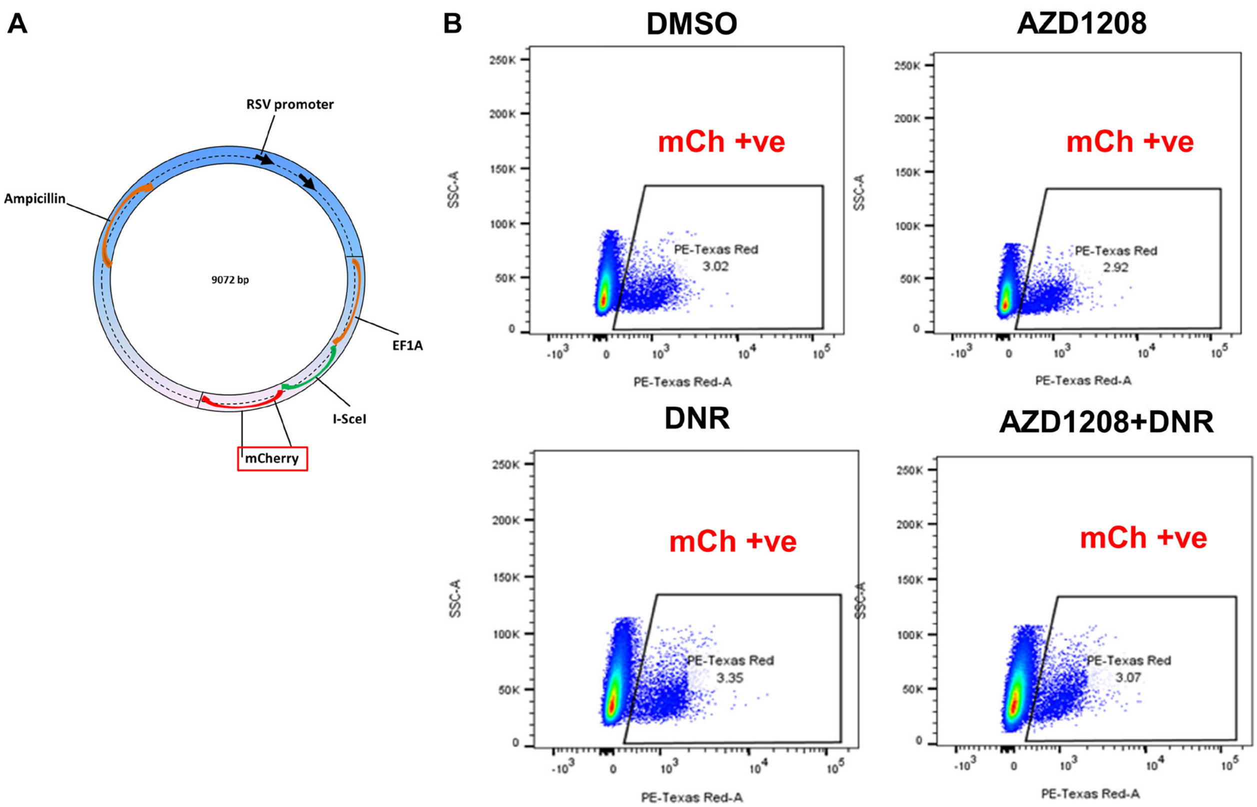

Supplementary Figure 1: (**A**) Schematic of I-SceI plasmid used for preparing I-SceI lentivirus. (**B**) Drug treatments do not alter expression of I-SceI post lentiviral transduction. Ba/F3-ITD cells with the DR-GFP repair construct were transduced with I-SceI lentivirus and treated with DNR and/or AZD1208 or DMSO control. Percentages of mCherry-positive (mCh+) cells were analyzed on a BD LSR II 36 hours post treatments. Representative figures are shown. Similar results were obtained for cells expressing the two other repair reporters (data not shown).

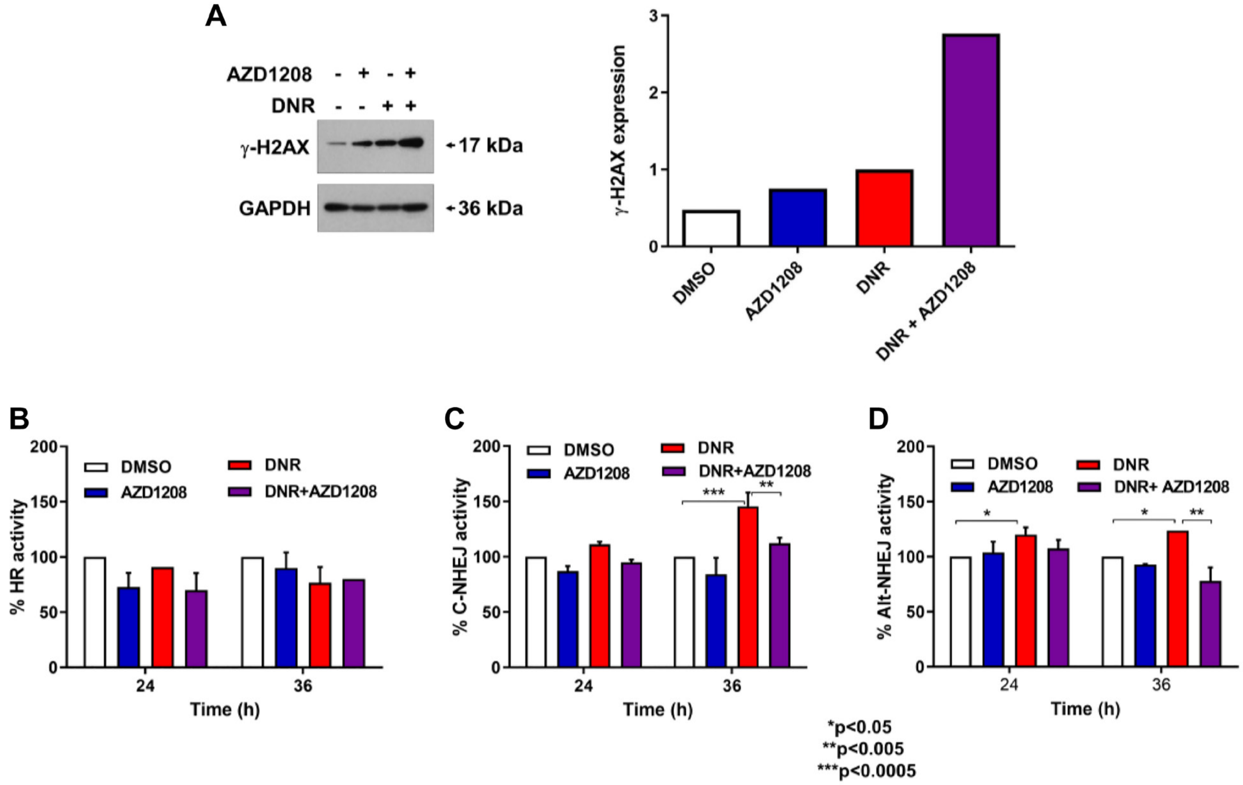

Supplementary Figure 2: Topoisomerase 2 inhibitor treatment induces both C-NHEJ and Alt-NHEJ repair activity in cells with FLT3-WT, abrogated by Pim kinase inhibitor co-treatment. (**A**) Concurrent treatment with Pim kinase inhibitor and topoisomerase 2 inhibitor DNR increases DNA double-strand breaks. Ba/F3-WT cells were treated with DNR and/or AZD1208. Whole cell lysates were immunoblotted with γ-H2AX and GAPDH primary antibodies. A representative immunoblot is shown. Densitometric analysis of two independent experiments is shown and percentage expression was plotted relative to pre-treatment levels, defined as 100%. (**B–D**) Pim kinase inhibition abrogates topoisomerase 2 inhibitor-induced increase in C-NHEJ and Alt-NHEJ activity. Ba/F3-WT cells with stable integration of DNA DSB reporters were transduced with I-SceI lentivirus and treated with 10 nM DNR and/or 1 μM AZD1208 to measure (B) HR, (C) C-NHEJ and (D) Alt-NHEJ repair activity. Percentages of GFP+ cells measured by flow cytometry were plotted relative to pre-treatment levels, defined as 100%. Means + S.E.M. of triplicate experiments are shown. $^{*}P < 0.05$, $^{**}P < 0.005$, $^{***}P < 0.0005$.

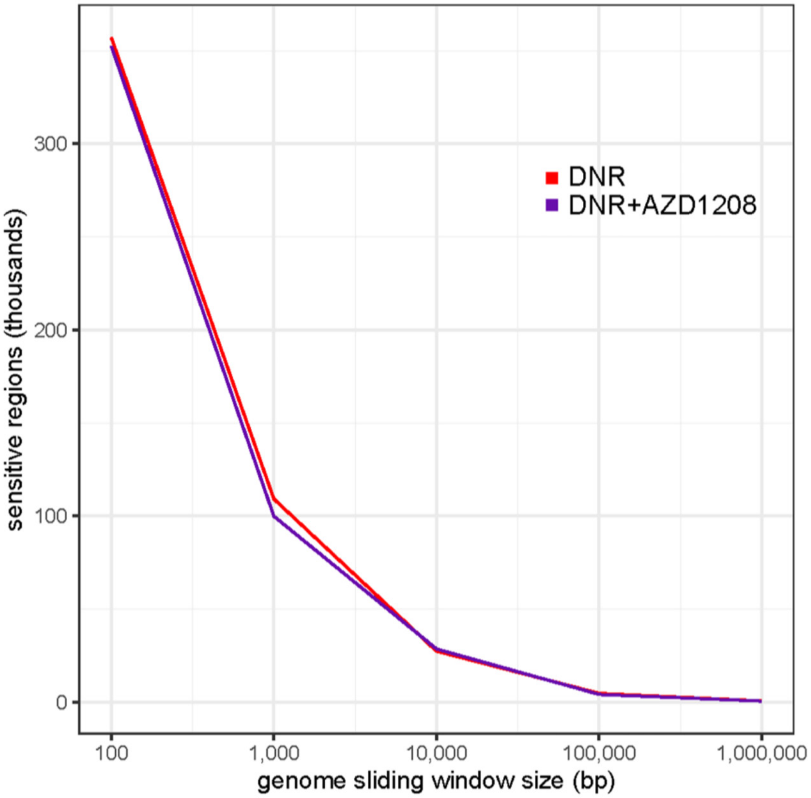

Supplementary Figure 3: Detection of enriched drug-induced DSBs at different genome window resolutions. MV4-11 DSB-seq read counts were calculated for multiple genome sliding window sizes (100 bp–1 Mbp). Drug-sensitive regions were characterized as genome windows having enriched DSB-seq read counts in the treatment condition (daunorubicin alone or co-treatment with AZD1208) relative to DMSO. Enrichment values were calculated according to the hypergeometric probability distribution and corrected for multiple hypotheses with the Benjamini-Hochberg method.

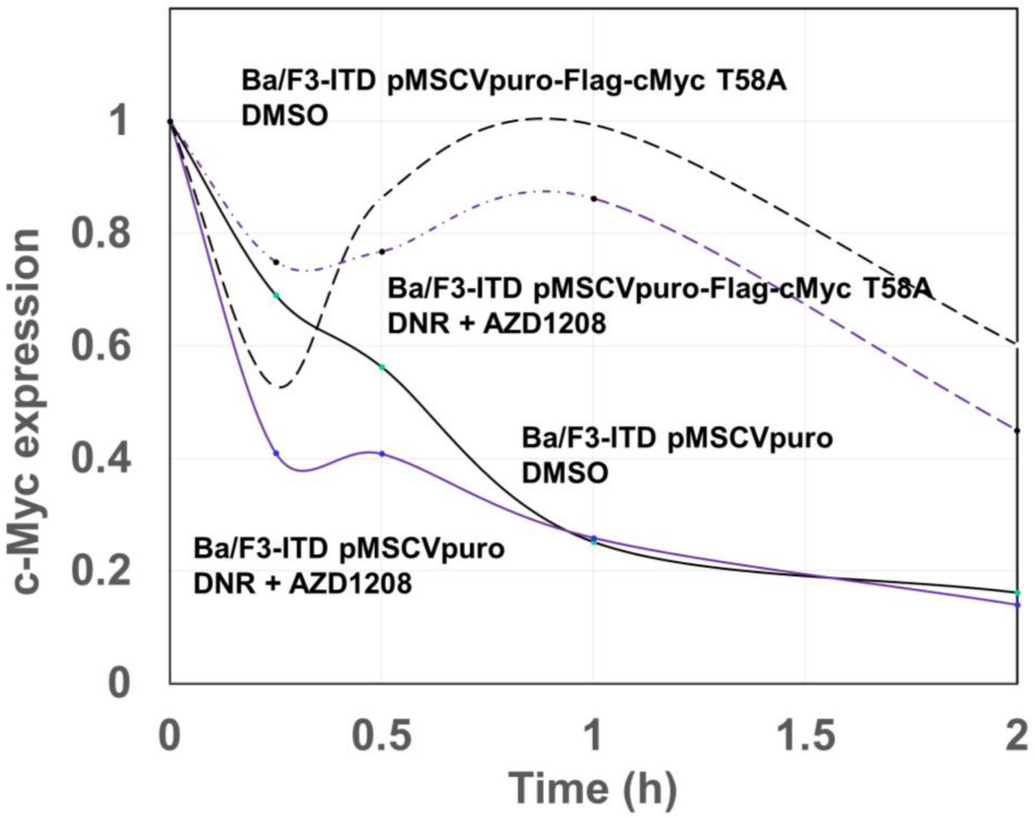

Supplementary Figure 4: c-Myc expression at serial time points in Ba/F3-ITD infected with cMyc T58A plasmid or empty vector control treated with daunorubicin and AZD1208 or DMSO control. c-Myc expression was normalized to vinculin expression at each time point. This is a graphical representation of the data in Figure 4E.

Supplementary Table 1: Read composition and mapping rates of DSB-seq datasets

Treatment	Selection	Paired reads	Bases sequenced	HISAT2 alignment %	LIMS Library ID
**DMSO**	None	86,849,814	14,992,925,309	3.17	IL100159780
**DMSO**	DSB	58,090,037	12,787,894,997	42.30	IL100159781
**DNR**	None	61,383,383	11,902,602,607	0.96	IL100159782
**DNR**	DSB	66,779,590	13,184,463,755	16.22	IL100159783
**AZD1208**	None	65,218,410	11,303,655,259	3.45	IL100159784
**AZD1208**	DSB	62,246,319	11,113,356,745	19.39	IL100159785
**DNR+AZD1208**	None	57,259,945	10,219,333,614	2.11	IL100159778
**DNR+AZD1208**	DSB	62,821,559	13,423,437,097	32.52	IL100159779

Supplementary Table 2: Patient samples

Patient	Age/Sex	WBC	% blasts	Karyotype	Mutations	FLT3-ITD size*
1	73F	96.8	46	46,XX	FLT3-ITD (54%), RUNX1, ASXL1, SF3B1	358.77
2	63F	28.6	84	46,XX	FLT3-ITD x 2 (73.4%, 14.3%)	414.04<br>349.91

*Base pairs.
